# Linking cervicovaginal immune signatures, HPV and microbiota composition in cervical carcinogenesis in non-Hispanic and Hispanic women

**DOI:** 10.1038/s41598-018-25879-7

**Published:** 2018-05-15

**Authors:** Paweł Łaniewski, Dominique Barnes, Alison Goulder, Haiyan Cui, Denise J. Roe, Dana M. Chase, Melissa M. Herbst-Kralovetz

**Affiliations:** 1 Department of Basic Medical Sciences, College of Medicine-Phoenix, University of Arizona, Phoenix, AZ USA; 2 Maricopa Integrated Health Systems, Phoenix, AZ USA; 3 Dignity Health St. Joseph’s Hospital and Medical Center, Phoenix, AZ USA; 4 UA Cancer Center, University of Arizona, Tucson/Phoenix, AZ USA; 5 Department of Obstetrics and Gynecology, College of Medicine-Phoenix, University of Arizona, Phoenix, AZ USA; 6 US Oncology, Phoenix, AZ USA

## Abstract

While high-risk human papillomavirus (HPV) infection is a well-established risk factor for cervical cancer, there are likely other factors within the local microenvironment that contribute to cervical carcinogenesis. Here we investigated relationships between HPV, vaginal pH, vaginal microbiota (VMB) composition, level of genital immune mediators and severity of cervical neoplasm. We enrolled women with low- and high-grade cervical dysplasia (LGD, HGD), invasive cervical carcinoma (ICC), and healthy controls. HPV16, HPV45, HPV58, and HPV31 were the most prevalent in our cohort with HPV16 and HPV31 genotypes more prevalent in Hispanics. Vaginal pH was associated with ethnicity and severity of cervical neoplasm. *Lactobacillus* dominance decreased with the severity of cervical neoplasm, which correlated with elevated vaginal pH. Hispanic ethnicity was also associated with decreased *Lactobacillus* dominance. Furthermore, *Sneathia* was enriched in all precancerous groups, ICC, abnormal pH and Hispanic origin. Patients with ICC, but not LGD and HGD, exhibited increased genital inflammatory scores and elevated specific immune mediators. Notably, IL-36γ was significantly associated with ICC. Our study revealed local, host immune and microbial signatures associated with cervical carcinogenesis and provides an initial step to understanding the complex interplay between mucosal inflammation, HPV persistence and the VMB.

## Introduction

Cervical cancer is the most common human papillomavirus (HPV)-related malignancy with estimated 526,000 new cases and 239,000 deaths worldwide in 2015^1^. Notably, incident rates of cervical cancer vary among different racial and ethnic groups^2,3^. In the United States, cervical cancer is more prevalent in Hispanic and African American women, compared to other races or ethnicities^3^. This disproportionate burden of cervical cancer in these populations has been primarily attributed to a lack of screening and an unequal access to health care; however, other ethnic-related factors might also contribute to these health disparities.

Although persistent infection with high-risk (HR) HPV genotypes is the primary cause of precancerous cervical intraepithelial neoplasia (CIN) and invasive cervical carcinoma (ICC), only a small portion of women infected with HPV progress to CIN, and if not treated, to ICC^4^, suggesting that other factors in the local microenvironment in conjunction with HPV play a role in cancer progression. An epidemiologic study associated *Chlamydia trachomatis* with the development of ICC^5^. We and others hypothesize a mechanistic role of vaginal microbiota (VMB) in both HPV persistence and cervical cancer progression^6–15^.

Lactobacilli protect the host against genital infections by producing lactic acid (which lowers vaginal pH below 4.5), secretion of antimicrobial compounds and competitive exclusion^16–19^. The VMB composition of reproductive age women is *Lactobacillus*-dominated (LD) with four predominant species (i.e., *Lactobacillus crispatus*, *Lactobacillus gasseri*, *Lactobacillus iners* or *Lactobacillus jensenii*), and this LD has been associated with vaginal health^16–19^. However, in some women, the VMB lacks a high proportion of lactobacilli and is dominated by a diverse mixture of anaerobic and microaerophilic bacteria (e.g., *Gardnerella*, *Atopobium*, *Prevotella*, *Sneathia*) commonly associated with bacterial vaginosis (BV). VMB composition varies between women of different ethnic/racial groups with higher prevalence of non-*Lactobacillus*-dominant (NLD) VMB in African American and Hispanic women^16,20,21^. However, the higher frequency of NLD VMB in these populations may reflect higher rates of asymptomatic BV^19^, and controlling for BV eliminates these differences^22^.

Several epidemiological studies reported an association between BV and HPV infection and persistence^8–10^. Women infected with HPV without cervical disease have been shown to exhibit more diverse VMB relative to HPV-negative women^6,7^. Two longitudinal studies also revealed that *L. gasseri*-dominant VMB was associated with HPV clearance, whereas enrichment in *Atopobium* spp. was associated with HPV persistence^11,15^. Two additional reports showed increasing VMB diversity in patients with CIN and ICC^12,13^. However, both studies had a very limited number of ICC cases and authors recommended larger cohort studies to validate their findings. In addition, these studies identified *Sneathia* spp. to be associated with HPV detection and/or cervical neoplasm^12,13,15^.

Furthermore, BV-associated bacteria have been associated with genital inflammation, specifically increased levels of proinflammatory cytokines, but not cervical immune cells. *Lactobacillus* dominance, particularly *L. crispatus*, has been associated with low levels of genital inflammation^23–29^. Inflammatory profiles differ between BV and classical sexually transmitted diseases such as gonorrhea, chlamydia or genital herpes^25,28^. Nevertheless, chronic genital inflammation may promote carcinogenesis similar to other mucosal sites. In clinical studies, HPV infection or clearance has not been associated with increased levels of genital inflammation, but rather VMB composition^30,31^. However, one study showed increased levels of proinflammatory cytokines in patients with cervical dysplasia^32^.

Herein, we performed a cross-sectional study in Arizona Hispanic and non-Hispanic women to identify associations between HPV genotype distribution, vaginal pH, VMB composition, levels of genital immune mediators, ethnicity and severity of cervical neoplasm. Our systematic approach allowed us to identify unique host immune and microbial signatures associated with cervical carcinogenesis in our cohort. To our knowledge this is the first report comprehensively exploring the complex interplay between HPV, VMB, genital immune mediators, disease progression and patient-specific factors such as ethnicity.

## Results

### Patient demographics

A total of 100 premenopausal women were recruited, enrolled in the study and classified into five groups: HPV-negative controls (Ctrl HPV−; n = 20), HPV-positive controls (Ctrl HPV+; n = 31), low-grade dysplasia (LGD; n = 12), high-grade dysplasia (HGD; n = 27) and invasive cervical carcinoma (ICC; n = 10). Table 1 shows the demographics. There was no significant difference among the groups in terms of age (*P* = 0.64), body mass index (BMI) (*P* = 0.96), HPV status [including HPV16, HPV18, high-risk (HR) and low-risk (LR) genotypes distribution] (*P* > 0.05), HPV risk profile (including infections with single or multiple HR genotypes as well as mixed infections with HR and LR genotypes) (*P* > 0.10), gravidity (*P* = 0.25), parity (*P* = 0.51) or previous loop electrosurgical excision procedure (LEEP) (*P* = 0.27). Forty-seven percent of patients were of Hispanic origin and 53% were of non-Hispanic origin. Hispanic ethnicity was not significantly different among the groups, ranging from 25.0% (5/20) in Ctrl HPV− to 63.0% (17/27) in HGD (*P* = 0.11). Two factors, pH and type of contraception, varied across the groups. The rates of hormonal contraceptive use were significantly different (*P* = 0.02) with the lowest rate in Ctrl HPV− (18.2%; 2/9) and the highest rate in ICC (80.0%, 4/5). However, contraception data were only available for 62% (62/100) of the women.

**Table 1:** Participant demographics. pH data available for 94 individuals; contraception data available for 62 individuals; gravidity and parity data available for 93 individuals; previous loop electrosurgical excision procedure (LEEP) data available for 86 individuals.

	n	Ctrl HPV−(n = 20)	Ctrl HPV+ (n = 31)	LGD (n = 12)	HGD (n = 27)	Cancer (n = 10)	*P* value
Age (mean (SD))	100	39.55 (7.35)	37.64 (9.38)	35.08 (7.24)	38.29 (8.46)	38.90 (9.09)	0.64
Ethnicity (n (%))							
Hispanic	47	5 (25.00)	14 (45.16)	7 (58.33)	17 (62.96)	4 (40.00)	
Non-Hispanic	53	15 (75.00)	17 (54.84)	5 (41.67)	10 (37.04)	6 (60.00)	0.11
pH (n (%))							
≤4.5	20	9 (45.00)	6 (22.22)	3 (27.27)	2 (7.41)	0 (0.00)	
>4.5	74	11 (55.00)	21 (77.78)	8 (72.73)	25 (92.59)	9 (100.00)	0.01
BMI (n (%))							
≤25	34	7 (35.00)	12 (38.71)	4 33.33)	8 (29.63)	3 (30.00)	
>25	66	13 (65.00)	19 (61.29)	8 (66.67)	19 (70.37)	7 (70.00)	0.96
Type of contraception							
Hormonal: yes	22	2 (18.18)	11 (52.38)	3 (42.86)	2 (11.11)	4 (80.00)	0.02
Hormonal: no	27	4 (36.36)	8 (38.10)	2 (28.57)	12 (66.67)	1 (20.00)	
No contraception	13	5 (45.45)	2 (9.52)	2 (28.57)	4 (22.22)	0 (0.00)	
HPV status							
HPV16 positive			17 (54.84)	8 (72.73)	19 (70.37)	7 (70.00)	0.59
HPV18 positive			0 (0.00)	1 (9.09)	4 (14.81)	1 (10.00)	0.11
Other high-risk			18 (58.06)	10 (83.33)	19 (70.37)	4 (40.00)	0.16
Low-risk			5 (16.13)	0 (0.00)	1 (3.70)	1 (10.00)	0.26
HPV risk profile							
Single high-risk			14 (45.16)	3 (25.00)	13 (48.15)	7 (70.00)	0.23
Multiple high-risk			12 (38.71)	8 (66,67)	13 (48.15)	2 (20.00)	0.16
High- and low-risk			26 (83.87)	11 (91.67)	26 (96.30)	9 (90.00)	0.46
Gravidity (n (%))							
0	15	3(15.00)	6 (21.43)	1 (8.33)	1 (4.17)	4 (44.44)	0.25
1–4	58	14 (70.00)	15 (53.57)	8 (66.67)	18 (75.00)	3 (33.33)	
5+	20	3 (15.00)	7 (25.00)	3 (25.00)	5 (20.83)	2 (22.23)	
Parity (n (%))							
0	20	4 (20.00)	8 (28.57)	1 (8.33)	3 (12.50)	4 (44.44)	0.51
1–4	61	14 (70.00)	17 (60.71)	8 (66.67)	18 (75.00)	4 (44.44)	
5+	12	2 (10.00)	3 (10.72)	3 (25.00)	3 (12.50)	1 (11.12)	
Previous LEEP (n (%))							
No	68	13 (81.25)	19 (70.37)	10 (100.00)	20 (83.33)	6 (66.67)	0.27
Yes	18	3 (18.75)	8 (29.63)	0 (0.00)	4 (16.67)	3 (33.33)	

*P* values calculated using ANOVA for continuous variables and Fisher’s exact test for categorical variables.

### Increased vaginal pH correlates with severity of cervical neoplasm and Hispanic ethnicity

**Figure 1:**
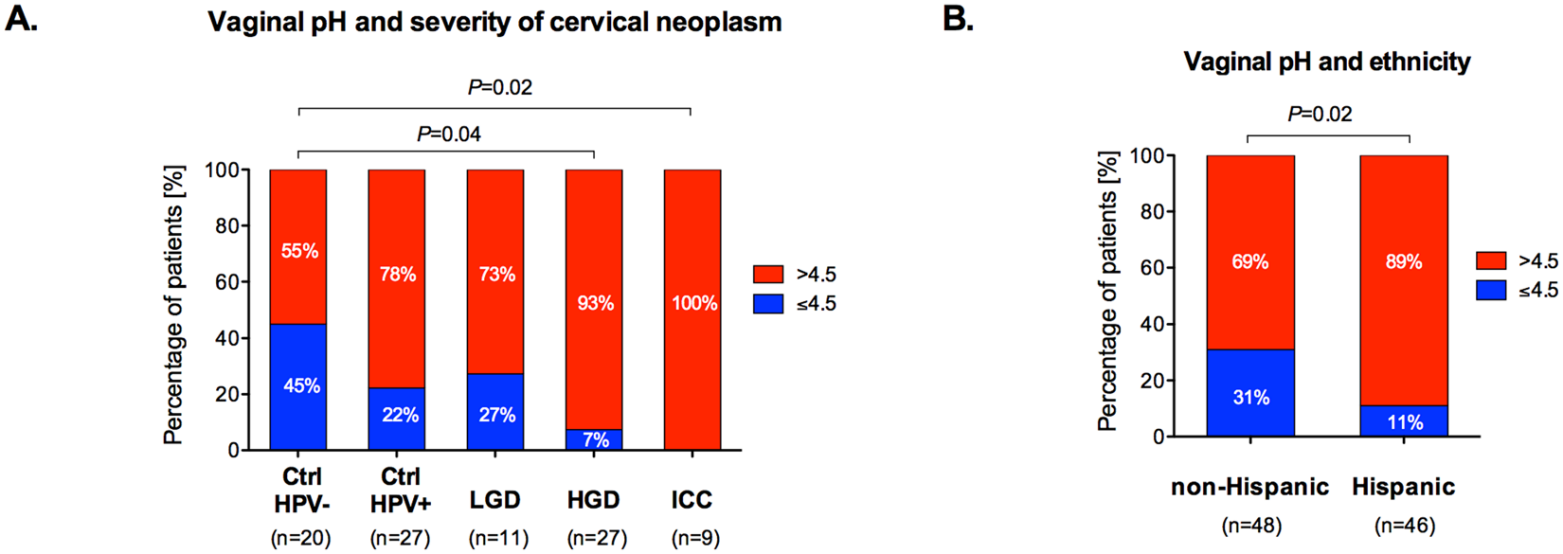
Vaginal pH significantly increases with severity of cervical neoplasm and is overall higher in Hispanic women. Percentage of patients with normal pH (≤4.5) and abnormal pH level (>4.5) among the groups (**A**) and across the groups in Hispanic and non-Hispanic women (**B**). *P* values calculated using Fisher’s exact test.

Vaginal pH was collected from 94% (94/100) of the women using nitrazine paper tests. Normal vaginal pH was defined as ≤4.5, and abnormal vaginal pH was defined as >4.5. Number of patients with abnormal pH significantly varied (*P* = 0.01, Table 1) and gradually increased among the groups (Fig. 1A**)**. Ctrl HPV− group had the lowest rate of abnormal pH (55.0%, 11/20). Then, percentage of patients with abnormal pH increased to 77.8% (21/27) and 72.7% (8/11) in Ctrl HPV+ and LGD, respectively. The highest rates of abnormal pH were observed in HGD and ICC, respectively, 92.6% (25/27) and 100% (9/9), which was a significant increase when compared to Ctrl HPV− group. The differences in the percentage of women with normal and abnormal vaginal pH across groups remained statistically significant after adjustment for age and BMI (*P* = 0.003) (Supplementary Table S1). Moreover, vaginal pH (normal vs. abnormal) was not significantly associated with gravidity or parity (Supplementary Fig. S1). Mean pH values were also significantly different among the groups (*P* = 0.006) (Supplementary Fig. S2A). This analysis showed that vaginal pH level was increased at various stages of cervical carcinogenesis and abnormal vaginal pH is highly associated with cancer. Interestingly, analysis of vaginal pH across the groups in Hispanic and non-Hispanic patients revealed that 89.1% (41/46) of Hispanic women exhibit abnormal pH compared to 68.8% (33/48) of non-Hispanic women (*P* = 0.02) (Fig. 1B, Supplementary Fig. 2B). However, the difference in the percentage of women with normal and abnormal vaginal pH between ethnicities was attenuated after adjusting for age and BMI (*P* = 0.07), but it was still significant after adjusting only for age (*P* = 0.03) or only for BMI (*P* = 0.048).

### HPV genotype distribution in our cohort is in accordance with the general population

**Figure 2:**
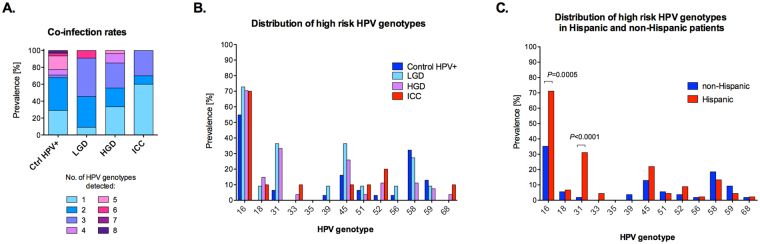
Prevalence of HPV genotypes in our cohort. Frequency of single and multiple HPV infections among the groups (**A**). Prevalence of 13 high-risk HPV genotypes detected among the groups (**B**) and ethnicities (**C**). *P* values calculated using Fisher’s exact test.

We determined the distribution of 37 HR and LR HPV genotypes in our cohort. The analysis showed that among HPV-positive women, 46.8% (37/79) of individuals were infected with single HR HPV genotype, 44.3% (35/79) were infected with multiple HR HPV genotypes, and only 1.3% (7/79) were infected with LR HP genotypes (Table 1). Furthermore, 91.1% (72/79) of women were positive for both HR and LR HPV genotypes (Table 1). The number of HPV genotypes detected ranged from 1 to 8 in each individual sample and the proportion of single and multiple type infections significantly varied among the groups (*P* = 0.02) (Fig. 2A). The most prevalent HPV genotypes in our cohort were HPV16 (64.6%, 51/79), HPV45 (21.5%, 21/79), HPV58 (20.3%, 16/79) and HPV31 (18.9%, 15/79) (Fig. 2B). While Ctrl HPV+ patients were co-infected with up to 8 different HPV genotypes (mean = 2.68 ± 1.83), the maximum number of concurrent HPV genotypes detected in ICC samples was only three (mean = 1.70 ± 0.94). This analysis showed that the distribution of HPV genotypes in our cohort was similar to larger epidemiologic studies^33,34^. Interestingly, when we analyzed the HPV distribution across the groups with regard to ethnicity, prevalence of two HR HPV genotypes was significantly higher in Hispanic compared to non-Hispanic patients. The percentage of HPV16 was 82.05% (32/39) in Hispanic compared to 47.5% (14/40) in non-Hispanic (*P* = 0.002), whereas percentage of HPV31 was 35.9% in Hispanic (14/39) compared to 2.5% (1/40) in non-Hispanic (*P* = 0.0001) (Fig. 2C). These data reveal higher prevalence of two HR HPV genotypes in Hispanic compared to non-Hispanic women.

### High microbiome diversity and *Lactobacillus* depletion is associated with increased vaginal pH and correlates with the severity of cervical neoplasm

**Figure 3:**
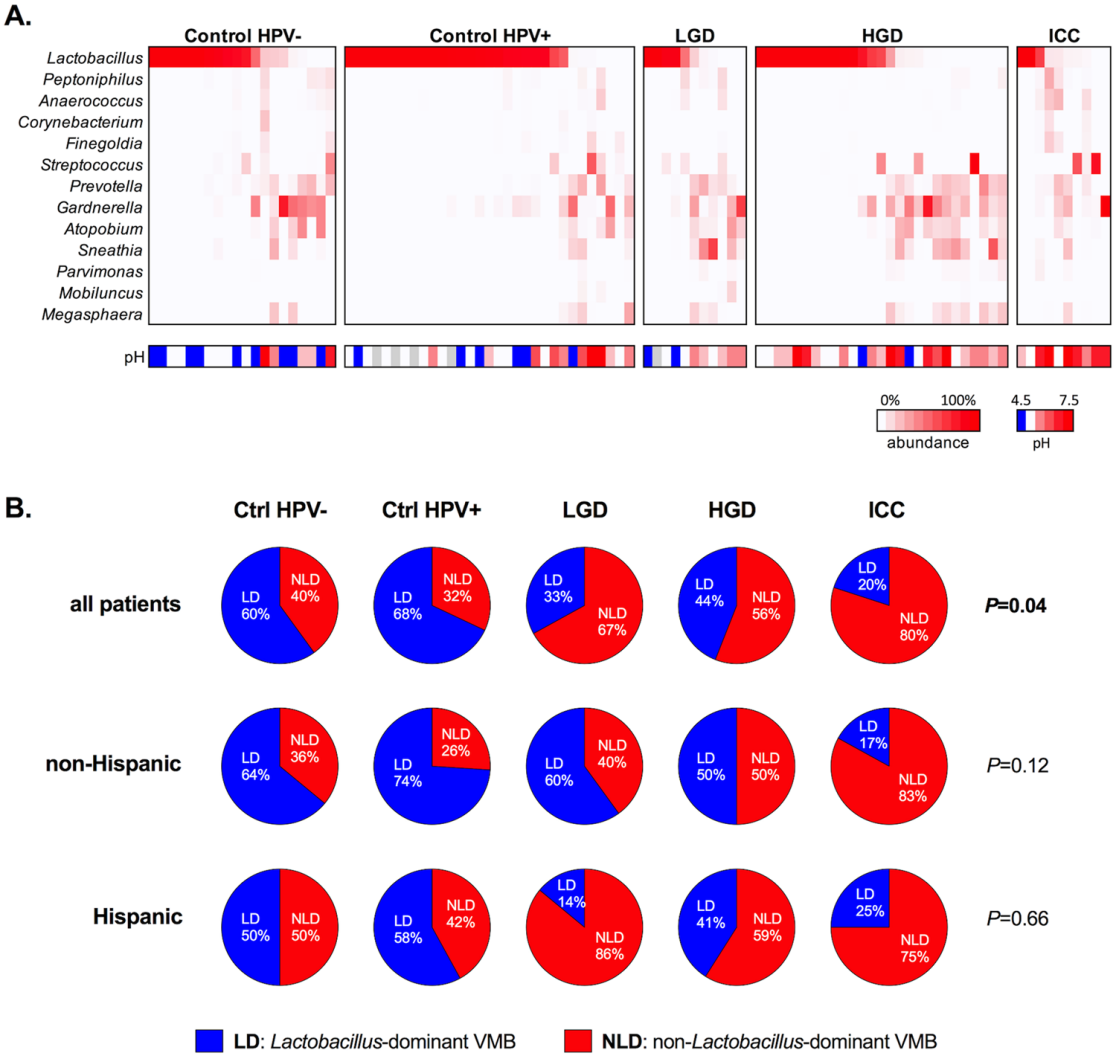
Vaginal microbiota (VMB) composition changes to more diverse and less *Lactobacillus*-dominant with severity of cervical neoplasm that correlates with increased vaginal pH. Heat map reflects relative abundance of the most prevalent bacterial genera and pH level (**A**). Percentage of patients with *Lactobacillus*-dominant (LD) and non-*Lactobacillus*-dominant (NLD) VMB among the groups and ethnicities (**B**). *P* values calculated using Fisher’s exact test.

**Figure 4:**
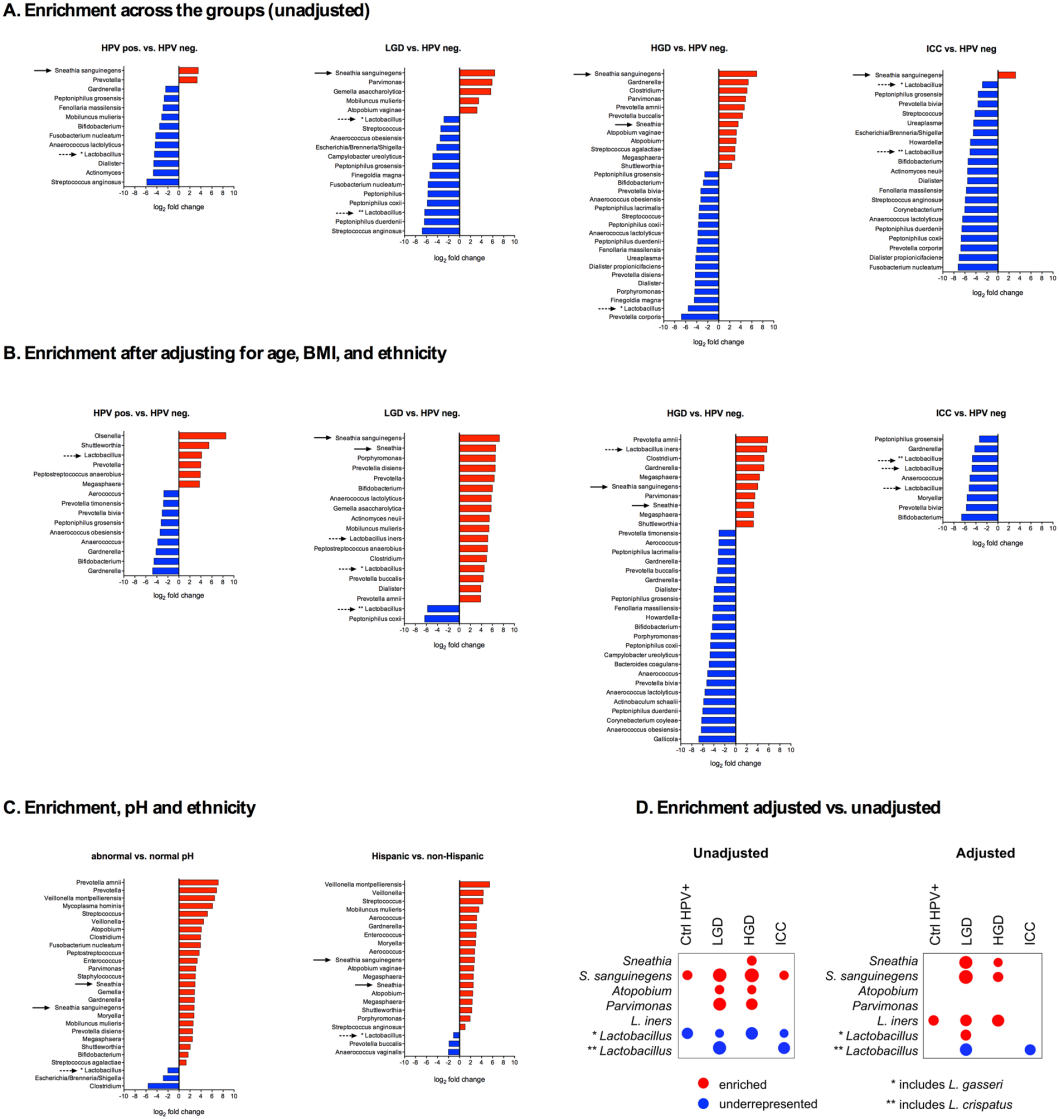
*Lactobacillus* is underrepresented whereas *Sneathia* is enriched in HPV-positive, LGD, HGD and ICC samples when compared to HPV-negative controls as well as in patients with abnormal pH and Hispanic women across the groups. Enrichment in bacterial taxa among the groups when compared to HPV-negative control prior (**A**) and after adjusting for age, BMI, and ethnicity (**B**). Enrichment in bacterial taxa in patients with abnormal pH vs. normal pH and Hispanic vs. non-Hispanic (**C**). Comparison of enrichment before and after adjustment for covariates (**D**). The bacterial enrichment was calculated using the DESeq. 2 package. The threshold for log_2_ fold difference was 2. Overall differences were tested using a likelihood ratio test, with pairwise comparisons using the Benjamini-Yekutieli false discovery adjustment. Red and blue bars/circles indicate enrichment or underrepresentation of taxa compared to HPV-negative control. The size of circles indicates the fold difference compared to HPV-negative control. Arrows indicate enrichment or underrepresentation in *Lactobacillus* and *Sneathia* spp.

To examine the composition of VMB in patients at various stages of cervical carcinogenesis, we performed sequencing of the variable region 4 (V4) of 16S rRNA gene. The VMB analysis revealed that vaginal pH, ethnicity and age were significantly correlated with β-diversity differences (Supplementary Table S2). Furthermore, the principal coordinate analysis showed that samples did not cluster in weighted ordination according to severity of cervical neoplasm, but did separate according to vaginal pH, confirming that vaginal pH levels correlate with changes in the VMB composition (Supplementary Figs S3 and S4**)**. Moreover, increasing severity of cervical neoplasm associated with decreasing relative abundance of *Lactobacillus* spp. and increasing abundance of a variety of microaerophiles and anaerobes (Fig. 3A). Rates of *Lactobacillus*-dominant (LD) VMB (defined as ≥80% relative abundance of *Lactobacillus* spp.) were significantly decreased, whereas dysbiotic non-*Lactobacillus*-dominant (NLD) VMB were increased in LGD (67%), HGD (56%) and ICC (80%) when compared to Ctrl HPV− (40%) or Ctrl HPV+ (32%) (*P* = 0.04) (Fig. 3B). We also observed that Hispanic patients overall exhibited a more diverse VMB compared to non-Hispanic patients (Fig. 3B). Since sequencing of V4 region is not sufficient to identify lactobacilli at the species level, we performed quantitative PCR analysis using primers specific to four predominant vaginal *Lactobacillus* species: *L. crispatus*, *L. gasseri*, *L. iners* and *L. jensenii*. The analysis revealed that *L. iners* and *L. crispatus* were the most prevalent lactobacilli in our cohort and the abundance of *L. crispatus*, but not other lactobacilli, was significantly increased in patients with HGD compared to Ctrl HPV+ (*P* = 0.01) (Supplementary Fig. S5). Furthermore, to identify differential taxa that may be related to increasing severity of cervical neoplasm, we tested for differences between the four patient groups compared to the HPV-negative control, using DESeq2 analysis^35–37^. We found that *Sneathia* spp. were significantly enriched, whereas *Lactobacillus* spp. were underrepresented in ICC, as well as, in the precancerous groups (LGD, HGD) and Ctrl HPV+ (Fig. 4A,D). Other BV-associated bacteria, *Atopobium* and *Parvimonas* were significantly enriched in both precancerous groups (LGD and HGD), whereas other BV-associated bacteria (e.g., *Gardnerella*, *Prevotella*, *Megasphaera*, and *Shuttleworthia*) were enriched only in HGD. To eliminate possible effect of confounders, we controlled our data for age, BMI and ethnicity. We did not adjust for pH, since increased vaginal pH directly correlated with changes in the VMB composition across our groups (Supplementary Figs S3 and S4). Following adjustment, we still observed enrichment in *Sneathia* spp. in both precancerous groups (LGD, HGD) (Fig. 4B,D). *Atopobium* and *Parvimonas* were not significantly enriched in any groups. Following adjustment, we also found *L. iners* to be enriched in HPV+, LGD and HGD groups. Interestingly, *Sneathia* and *Atopobium* were also enriched in women with abnormal pH or of Hispanic ethnicity, whereas *Lactobacillus* spp. were underrepresented in these women (Fig. 4C). This data indicate that *Sneathia* spp. or together with other BV-associated bacteria might play a role in cervical carcinogenesis.

### Patients with invasive carcinoma, but not precancerous neoplasm exhibit increased levels of genital immune mediators and genital inflammatory score

**Figure 5:**
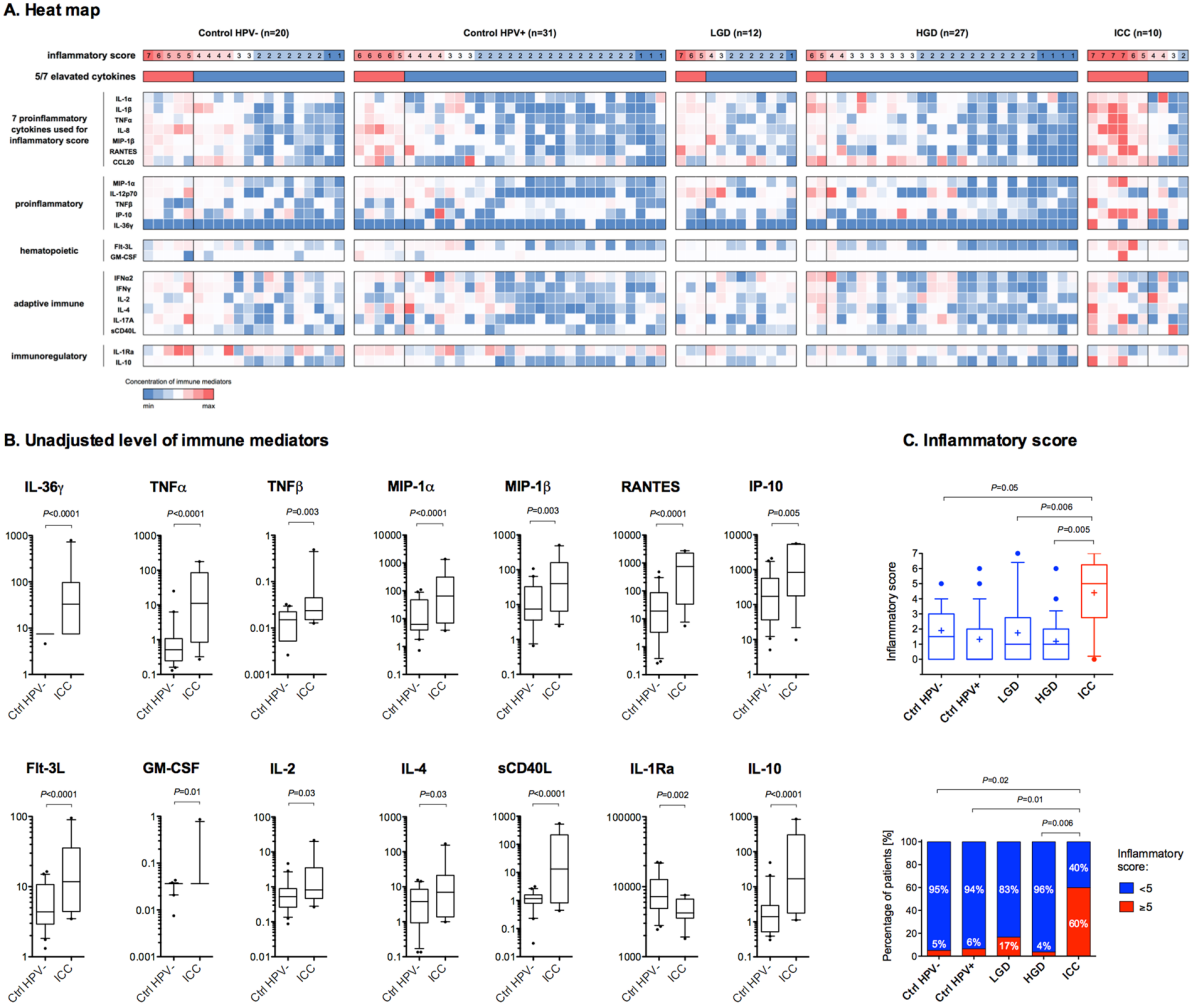
Patients with ICC exhibit significantly increased genital immune mediators and genital inflammation score. Heat map reflects relative levels of immune mediators across all the samples and inflammatory scores (**A**). Increasing brightness of red and blue indicate higher and lower concentration of each protein, respectively. Levels of immune mediators significantly altered in ICC when compared to Ctrl HPV− (**B**). Boxplot whiskers range between 10^th^ and 90^th^ percentiles, dots indicate outliers. *P* values were calculated using linear mixed effects models where group was the fixed effect and replicate was the random effect with Tukey adjustment. Level of inflammatory scores among the groups (*P* values calculated using Kruskal-Wallis test) and percentage of patients with genital inflammation, defined as having at least 5/7 of the following cytokines in the upper quartile: IL-1α, IL-1β, IL-8, MIP-1β, CCL20 (MIP-3α), RANTES, and TNFα (*P* values calculated using Fisher’s exact test) (**C**).

**Figure 6:**
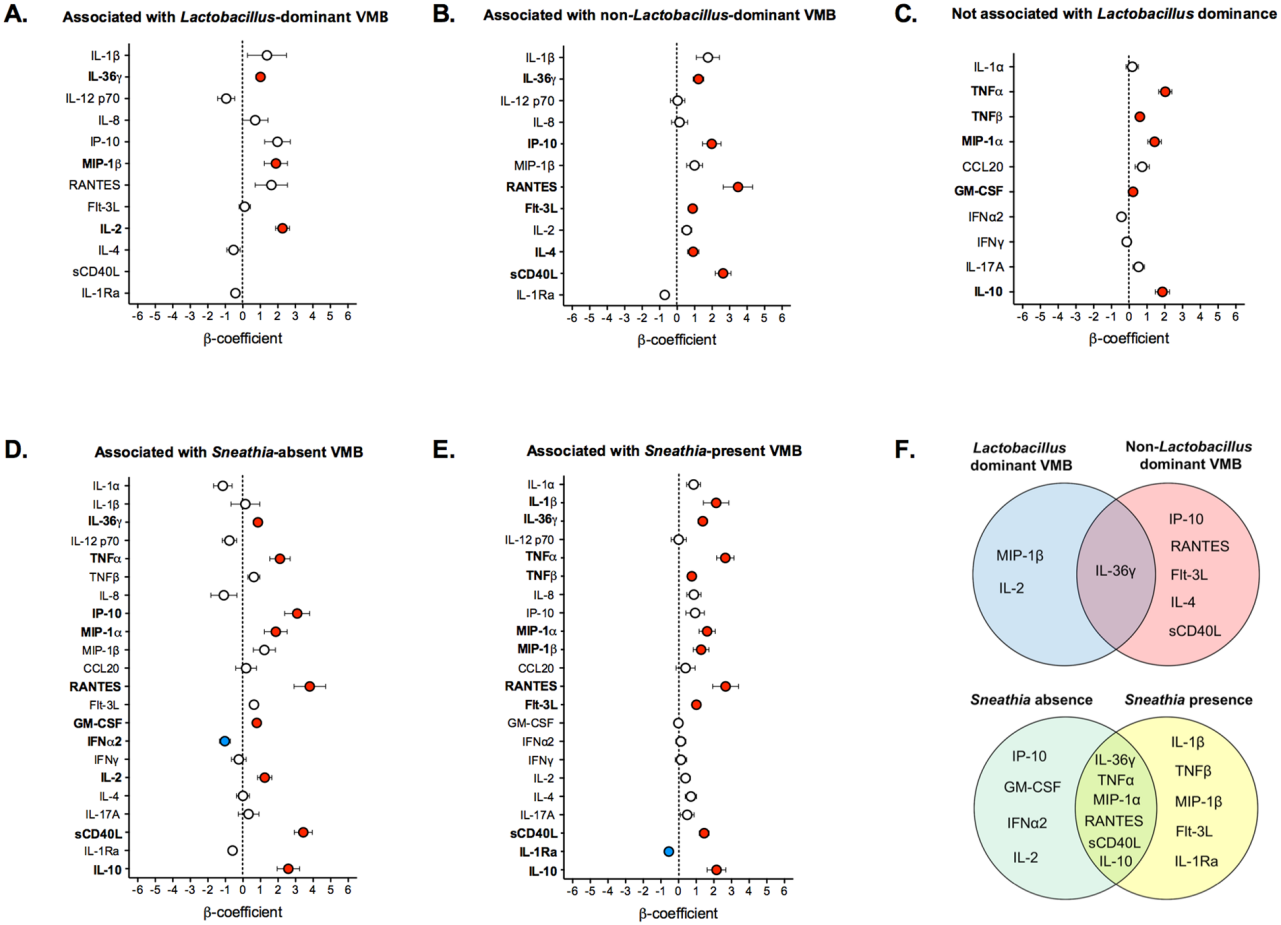
IL-36γ is significantly associated with ICC regardless of VMB after adjusting for age, BMI, ethnicity, and *Lactobacillus* dominance or *Sneathia* presence. β-coefficients of immune mediators that were not associated with (**A**) or associated with *Lactobacillus* dominance (defined as ≥80% relative abundance) (**B**,**C**). β-coefficients of immune mediators that were associated with *Sneathia* presence (defined as ≥0.01% relative abundance) (**D**,**E**). Dots indicate β-coefficients of linear regression analysis; error bars represent standard error (SE). Red and blue dots indicate positive or negative associations, respectively, that were significant compared to Ctrl HPV− after adjusting for covariates (*P* < 0.05). Venn diagram showing relations between VMB composition and associations of immune mediators with ICC and VMB composition (**F**).

To characterize local immune signatures in patients at various stages of cervical carcinogenesis, we measured levels of 22 immune mediators in cervicovaginal lavages (Fig. 5A). We found that seven proinflammatory and chemotactic cytokines: IL-36γ, TNFα, RANTES, MIP-1α, MIP-1β, RANTES, IP-10; two hematopoietic cytokines: Flt-3L, GM-CSF; three adaptive immune cytokines: IL-2, IL-4, sCD40L, and an anti-inflammatory cytokine IL-10, were significantly elevated in ICC, whereas the other immunoregulatory cytokine IL-1Ra was significantly reduced in ICC when compared to Ctrl HPV− (Fig. 5B). Within the ICC patients, there were also significant differences in levels between stage I patients vs. stage II-IV patients (Supplementary Fig. S6). None of the immune mediators tested were significantly increased in precancerous groups. The level of IFNγ was significantly decreased in Ctrl HPV+ and HGD and level of IL-1Ra was also lower in HGD (Supplementary Fig. S7). To reduce concerns about multiple comparisons, we utilized a previously described genital inflammation scoring system to show evidence of genital inflammation in our cohort^28,31,38^. Levels of seven cytokines (IL-1α, IL-1β, IL-8, MIP-1β, CCL20 (MIP-3α), RANTES, and TNFα) were used to determine inflammatory scores; patients were assigned one point for each mediator when the level was in the upper quartile. Using this scoring system, ICC, but not other precancerous groups, exhibited significantly elevated inflammatory score compared to Ctrl HPV− (*P* < 0.05) (Fig. 5C). As confirmation, we performed a principal components analysis on the 7 cytokines (Supplementary Fig. S8). The first principal component explained 59% of the variance. There was a statistically significant overall difference in the first principal component scores across the 5 severity groups (*P* = 0.0003). Pairwise comparisons demonstrated statistically significant differences between the Ctrl HPV− and ICC (*P* = 0.007), Ctrl HPV+ and ICC (*P* = 0.0003), and HGD and ICC (*P* = 0.0002). Similar differences were observed also after adjusting for age, BMI and ethnicity. Then, we tested various scoring systems for evaluation of evidence of genital inflammation, which defined genital inflammation as having 3/7, 4/7, 5/7, 6/7 and 7/7 cytokines in the upper quartile (Supplementary Fig. S8). Using 5/7 scoring system was the most sensitive to show evidence of genital inflammation in our patients (see the trend analysis in Supplementary Fig. S8), and also reflected elevated levels of other proinflammatory cytokines and chemokines tested, but not included in the score (Fig. 5A,C). Using this system, we showed that evidence of genital inflammation was significantly increased in ICC patients (60%) compared to Ctrl HPV− (5.0%; *P* = 0.02), Ctrl HPV+ (6.5%; *P* = 0.01) and HGD (3.7%; *P* = 0.006). Evidence of genital inflammation in other groups was not significantly increased compared to Ctrl HPV− (Fig. 5C). Since depletion of lactobacilli and enrichment in *Sneathia* correlated with the severity of cervical neoplasm, we examined associations between secreted immune mediators and *Lactobacillus* dominance (relative abundance ≥80%) or *Sneathia* presence (relative abundance ≥0.01%) (Supplementary Figs S9 and S10). For several of the mediators, the effect of the patient group differed in those with *Lactobacillus* dominance and *Sneathia* presence so stratified results are presented (i.e., there was a statistically significant interaction). Following adjustment for age, BMI and ethnicity, MIP-1β and IL-2 were positively associated with ICC in patients with LD VMB (Fig. 6A), whereas IP-10, RANTES, Flt-3L, IL-4 and sCD40L were positively associated with ICC in patients with NLD VMB when compared to Ctrl HPV− (Fig. 6B). IL-36γ was the only cytokine that was positively associated with ICC in patients with either LD or NLD VMB (Fig. 6F). On the other hand, TNFα, TNFβ, MIP-1α, GM-CSF and IL-10 were positively associated with ICC in all patients regardless of *Lactobacillus* dominance (Fig. 6C). All tested immune mediators were associated with *Sneathia* presence. After adjusting for covariates, IP-10, GM-CSF, and IL-2 were positively associated with ICC, whereas IFNα2 was negatively associated with ICC in patients with *Sneathia*-absent VMB when compared to Ctrl HPV− (Fig. 6D). IL-1β, TNFβ, MIP-1β and Flt-3L was positively associated with ICC, whereas IL-1Ra was negatively associated with ICC in patients with *Sneathia*-present VMB (Fig. 6E). IL-36γ, TNFα, MIP-1α, RANTES, sCD40L and IL-10 were positively associated with ICC in patients with either *Sneathia*-absent or present VMB (Fig. 6F). The analysis revealed unique immune signature associations between invasive carcinoma and VMB composition.

## Discussion

Persistent infection with HR HPV genotypes is the most important risk factor for cervical cancer precursors and ICC^4^. However, the cause of persistent HPV infection (as most infections are cleared) and progression to cervical cancer remain poorly understood^39^. In this report, we explored associations between HPV, vaginal pH, VMB composition, levels of secreted genital immune mediators and severity of cervical neoplasm to further investigate the complex interaction between these factors in the local microenvironment.

HPV genotype distribution in our cohort was similar to larger epidemiologic studies, as expected, with HPV16 being most prevalent (65% of HPV-positive samples)^33,34^. Other frequent genotypes detected included HPV45, HPV58 and HPV31. These HPV genotypes are targeted by the newest nonavalent HPV vaccine, but not the bi- and quadrivalent vaccines; however, the women in our cohort were not vaccinated. Infection with multiple genotypes were common. In addition, rates of co-infection with multiple genotypes decreased with the severity of neoplasm, in accordance with previous reports^40^. Interestingly, HPV16 and HPV31 were significantly more prevalent in Hispanic compared to non-Hispanic women. Others also reported racial/ethnic differences in HPV distribution among women with CIN^34,41,42^. In contrast to our data, Hariri *et al*. showed higher prevalence of HPV45 in Hispanic women and higher rates of HPV16/18-related lesions in non-Hispanic^34^. Notably, geographic differences in HPV distribution have been also reported^33,42^.

We found that abnormal vaginal pH (>4.5) correlated with the severity of cervical neoplasm. A large population-based study also showed that elevated vaginal pH is associated with oncogenic HPV detection, multiple HPV types infections and diagnosis of LGD^43^. Our data expand these findings showing positive association of abnormal pH with advanced cervical dysplasia and ICC. Walther-António *et al*. also reported high vaginal pH, combined with detection of *Atopobium vaginae* and *Porphyromonas* spp., to be associated with endometrial cancer^44^. Collectively, these data suggest that abnormal pH might be a marker of gynecologic cancer. Moreover, we also found that Hispanic women exhibited overall higher vaginal pH, in accordance with a previous report^16^. Elevated vaginal pH might be an additional risk factor for cervical cancer development/progression.

In addition, abnormal vaginal pH (>4.5) in our cohort correlated with decreased relative *Lactobacillus* abundance, in accordance with a previous report showing elevated vaginal pH in women with diverse NLD VMB^16^. In our study, NLD VMB significantly (*P* = 0.04) increased with severity of cervical neoplasm with significantly higher rates of diverse NLD VMB in patients with advanced cervical neoplasia and invasive carcinoma. A previous report also suggested that the lactobacilli depletion is associated with advancing CIN severity, but the analysis did not reach statistical significance^12^. Interestingly, we also observed that Hispanic women had higher ratios of diverse NLD VMB compared to non-Hispanic women. Higher rates of diverse NLD VMB in Hispanic women are consistent with a previous report^16^. However, the observed disproportion can be due to higher rates of asymptomatic BV^22^, which has been associated with HPV detection, as well as, delayed HPV clearance^8,10^. In contrast to other studies^6,7,11,15,31^, HPV positivity did not significantly alter the VMB composition in women without cervical dysplasia; however, the high proportion of Hispanic women in our cohort might contribute to this finding. Overall, our study indicates that diverse NLD VMB is also associated with cervical disease severity and progression.

With regard to specific bacterial taxa, the majority of identified bacteria that were enriched in HPV-positive women or women with LGD, HGD or ICC are commonly associated with BV (i.e., *Gardnerella, Atopobium*, *Prevotella*. *Megasphaera, Parvimonas, Peptostreptococcus, Anaerococcus, Sneathia*)^45^. However, we also identified bacterial genera associated with aerobic vaginitis (i.e. *Streptococcus agalactiae*) or other dysbioses (i.e*. Clostridium*) to be enriched in these women^46^. Moreover, we found novel bacterial taxa, i.e. *Shuttleworthia, Gemella* and *Olsenella*, to be also associated with HPV positivity and/or cervical neoplasia.

Interestingly, we found *Sneathia* spp. to be the only taxon that was significantly enriched in women infected with HPV, as well as, women with precancerous lesions and cervical cancer. Results of our study complement and extend previous findings^7,13,15^. Two previous studies showed higher levels of *Sneathia* in HPV-positive women compared to uninfected women and suggested that *Sneathia* might be a microbiological marker of HPV infection^7,15^. Mitra *et al*. also identified *S. sanguinegens* to be more prevalent in women with HGD compared to LGD^12^. Furthermore, Audirac-Chalifour *et al*. showed that *Sneathia* predominates in women with CIN, but not ICC^13^. Our study shows *Sneathia* to be associated with all stages of cervical carcinogenesis (i.e., Ctrl HPV+, LGD, HGD, ICC). Moreover, after controlling for known VMB confounders (age, BMI, ethnicity), our analysis revealed that precancerous LGD and HGD are enriched in *Sneathia*. This finding suggests that presence of *Sneathia* in VMB may also be a metagenomic marker for CIN progression. Lack of enrichment of *Sneathia* in invasive carcinoma following adjustment for covariates might be due to small ICC sample size. Alternatively, *Sneathia* might be outcompeted by other bacteria (“bacterial passengers”), having a competitive advantage in the tumor microenvironment, as hypothesized at other mucosal sites^47^.

Other novel taxon identified in this study to be associated with cervical dysplasia included *Prevotella amnii*, originally isolated from human amniotic fluid^48^. Previously, *Prevotella* spp. have been associated with HPV infection, but not identified to the species level^7^. We also found *Atopobium* and *Parvimonas* spp. to be enriched with low- and high-grade precancerous lesions. However, following adjustment, we did not detect this relationship, suggesting that enrichment in *Atopobium* and *Parvimonas* was associated with one of the covariates. Interestingly, following adjustment for covariates, we also found *L. iners* to be enriched in HPV-positive women, as well as, women with LGD and HGD. Others also reported *L. iners* to be associated with higher risk of cervical dysplasia^14,49^. *L. iners* can dominate VMB of healthy women; nevertheless, it is also found in the majority of women diagnosed with BV^50^. Moreover, *L. iners*-dominated VMB is more likely to transition to the dysbiotic VMB^51^. Our findings suggest that *L. iners* can contribute to the changes in VMB composition, which may lead to disease progression.

In this study, we systematically examined levels of secreted immune mediators in the cervicovaginal microenvironment at various stages of cervical carcinogenesis. Our analysis revealed increased levels of proinflammatory and chemotactic cytokines (IL-36γ, TNFα, RANTES, MIP-1α, MIP-1β, IP-10), hematopoietic cytokines (Flt-3L, GM-CSF), adaptive immune response cytokines (IL-2, IL-4, sCD40L), and an anti-inflammatory cytokine (IL-10) in women with ICC, but not with precancerous lesions. We did not observe an elevated proinflammatory response in HPV-positive women, which is in accordance with a recent report showing that neither HPV infection nor clearance is associated with broad differences in cervicovaginal cytokines^31^. We also evaluated a previously described inflammatory score system to avoid multiple comparisons^28,31,38^. We observed evidence of genital inflammation (defined by elevation of ≥5/7 cytokines) only in patients with ICC, but not with precancerous lesions, when compared to HPV-negative controls. Masson *et al*. showed that women with BV exhibited only moderately increased levels of pro-inflammatory cytokines, and decreased levels of chemotactic cytokines, whereas women with STI, such as chlamydia and gonorrhea, exhibit the highest levels of cervicovaginal cytokines^25^. We also did not observe an overt proinflammatory response in our STI-negative cohort, other than the cancer group. Shannon *et al*. used inflammatory scores and showed that only 45% women with the diverse NLD VMB demonstrate evidence of genital inflammation (defined by elevation of ≥3/7 cytokines). When we employed the same scoring system (≥3/7) for evidence of genital inflammation, we still did not observe any significant differences between precancerous groups and controls.

To decipher the relationship between VMB composition and immune mediators, we explored the associations between immune mediators and *Lactobacillus*, which are related to vaginal health, as well as, potentially pathogenic *Sneathia*. Several immune mediators, such as, TNFα, TNFβ, MIP-1α, GM-CSF and IL-10 were associated with cancer, but not *Lactobacillus*-dominance, suggesting that their elevated levels are cancer-driven. However, the associations of several other cytokines (IL-36γ, MIP-1β, RANTES, IP-10, IL-2, IL-4, Flt-3L, sCD40L) with cancer were dependent on the VMB composition. Interestingly, IL-36γ was highly associated with cancer, irrespective of VMB composition. IL-36γ, which belongs to the IL-1 family, is a proinflammatory cytokine expressed at various mucosal sites, including the female reproductive tract, and it functions as a putative “alarmin” in damaged tissue^52,53^. Our studies using a human 3-dimensional vaginal epithelial cell model showed that IL-36γ is a driver for epithelial and immune activation following microbial insult and might play a crucial role in the host defense against invading pathogens^52^. However, the role of IL-36γ in cancer is poorly understood. Wang *et al*. revealed that IL-36γ exerted anti-tumor effects *in vivo* and transformed the tumor microenvironment in favor of tumor eradication^54^. Moreover, the study showed that tumoral expression of IL-36γ in melanoma and lung cancer decreased tumor progression^54^. This might suggest that increased secretion of IL-36γ in the cervicovaginal microenvironment is a defense mechanism employed by the host to limit cervical cancer progression. On the other hand, in psoriasis, IL-36γ has been shown to promote the inflammatory response through the Wnt/β-catenin pathway, which is altered in many cancers^55^. This may suggest that IL-36γ is contributing to cervical carcinogenesis by promoting damaging inflammation. The impact of IL-36γ on cervical and other gynecologic cancers warrants further investigation.

A major strength of our study is a high proportion of Hispanic women, which allowed us to determine ethnic-specific factors contributing to health disparities. Compared to other reports, we systemically explored several factors (i.e. HPV, VMB, pH, ethnicity, level of secreted immune mediators), which may contribute to HPV persistence and/or disease progression. Moreover, we used sophisticated statistical analyses to adjust our microbiome and immune mediator data for known confounders. Our study has some limitations, including a relatively modest sample size and the lack of specific sexual behavior data, i.e. number of sexual partners or sexual debut age. We employed a cross-sectional study design; therefore, we focused on associations, not causation. Longitudinal studies are required to extend these findings and determine the extent to which a diverse NLD VMB or specific bacterial species are casual factors in cervical carcinogenesis or consequences of the disease.

In conclusion, we showed that vaginal pH was associated with ethnicity and significantly increased with the severity of cervical neoplasm. We also revealed that abnormal vaginal pH, Hispanic ethnicity and the severity of cervical neoplasm was significantly associated with depletion of lactobacilli. Furthermore, *Lactobacillus* dominance decreased with the severity of cervical neoplasm. We identified *Sneathia* spp., other BV-associated bacterial species and novel bacterial taxa to be associated with HPV infection and/or HPV-related cervical neoplasm. In addition, we found that women with ICC, but not women with precancerous lesions, exhibited evidence of genital inflammation. Notably, IL-36γ was identified as a unique immune mediator significantly associated with cervical cancer regardless of VMB composition. Overall, this study revealed unique immune and microbial signatures in the local microenvironment associated with cervical carcinogenesis in non-Hispanic and Hispanic women. Further studies, particularly those employing longitudinal designs, are required to better understand the molecular mechanisms involved in the complex role that bacterial communities and/or individual bacterial species may play in the development of cervical neoplasm and the progression to invasive disease.

## Methods

### Participant enrollment

Participants were consecutively recruited at three clinical sites located in Phoenix, AZ: St. Joseph’s Hospital, and Medical Center (SJHMC), University of Arizona (UA) Cancer Center and Maricopa Integrated Health System (MIHS) and screened based off eligibility criteria and approached to participate in the study. All patients provided informed written consent and all research and related activities involving human subjects were approved by the Institutional Review Boards at UA, SJHMC and MIHS and performed in accordance with federal guidelines and regulations. One hundred premenopausal, non-pregnant, women diagnosed with cervical dysplasia and ICC, as well as, healthy HPV-positive and HPV-negative women were enrolled and contributed to the study. Histology of colposcopy-directed biopsy samples was used to classify patients into groups. A two-tiered system of CIN was used: low-grade dysplasia (LGD) was defined as CIN1, and high-grade dysplasia (HGD) was defined as CIN2/3. If histology was not available (i.e. for healthy HPV-positive and HPV-negative controls), cytology was used for classification. Additionally, HPV status was determined by the Linear Array HPV Genotyping Tests (Roche, Indianapolis, IN), and used to subdivide healthy women into two groups: HPV-positive (Ctrl HPV+, including high- and low-risk HPV genotypes) and HPV-negative controls (Ctrl HPV−). Overall, patients were divided into five groups: women with LGD, women with HGD, women with ICC, including adenocarcinoma and squamous cell carcinoma, Ctrl HPV+ and Ctrl HPV−. Exclusion criteria for the study included: currently menstruating; currently on antibiotics, antifungals or antivirals or within the previous 3 months; current vaginal infection (including bacterial vaginosis), vulvar infection, urinary tract infection or sexually transmitted infection (chlamydia, gonorrhea, trichomoniasis, genital herpes) or within the previous 3 weeks; unusual or foul-smelling vaginal discharge; use of douching substances, vaginal applied medications and suppositories, feminine deodorant sprays, vaginal lubricants within 48 hours prior the visit; any skin condition in the genital area interfering with the study; sexual intercourse less than 48 hours prior the visit; type I or type II diabetes; hepatitis; being HIV-positive. The exclusion criteria were verified by physician’s pelvic exam, medical record and/or self-reported. The details of exclusion criteria are listed in the Supplementary Table S3.

### Sample collection

Vaginal swabs and cervicovaginal lavages (CVL) were collected by a clinician. A speculum was inserted without lubricant and two vaginal swabs were collected. The first swab was collected by swabbing the lateral walls of the mid vagina using an eSwab collection system containing Amies transport medium (COPAN Diagnostics, Murrieta, CA). The second swab was used to measure vaginal pH using nitrazine paper, and recorded by the clinician according to the manufacturer’s instructions using a scale of 4.5–7.5. Following vaginal swab collection, CVLs were collected using 10 ml of sterile 0.9% saline solution (Teknova, Hollister, CA). Following the collection, both vaginal swab and CVL were immediately placed on ice and frozen at −80 °C within 1 hour of collection.

### Sample processing

CVLs were thawed on ice and clarified by centrifugation (700 × *g* for 10 min at 4 °C). DNA was extracted from vaginal swabs using PowerSoil DNA Isolation Kit (MO BIO Laboratories, Carlsbad, CA) following the manufacturer’s instructions. Both CVL and extracted DNA were aliquoted to avoid freeze/thaw cycles and stored at −80 °C for further analysis.

### HPV genotyping

HPV genotyping was performed on DNA samples extracted from vaginal swabs using the Linear Array HPV Genotyping Tests (Roche) following the manufacturer’s instructions.

### Measurement of immune mediators in CVL

Levels of 21 cytokines and chemokines (IL-1α, IL-1β, IL-6, IL-12p70, TNFα, TNFβ, IL-8, IP-10, MIP-1α, MIP-1β, RANTES, Flt-3L, GM-CSF, IFNα2, IFNγ, IL-2, IL-4, IL-17A, sCD40L, IL-1Ra, IL-10) were determined in CVL aliquots using the Milliplex MAP Human Cytokine/Chemokine and Th17 Magnetic Bead Panels (Millipore, Billerica, MA) in accordance with the manufacturer’s protocol. Level of IL-36γ was measured in CVL aliquots by enzyme-linked immunosorbent assay using Human IL-36γ ELISA kit (RayBiotech, Norcross, GA) in accordance with manufacturer’s instructions. A four-parameter logistic regression curve fit was used to determine the concentration. All samples were assayed in duplicate.

### 16S rRNA sequencing analysis

16S rRNA sequencing analysis was performed by the Second Genome Inc. (San Francisco, CA). Briefly, the V4 region of bacterial 16S rRNA operon was amplified from the genomic DNA obtained from vaginal swabs and sequenced on the MiSeq platform (Illumina, San Diego, CA). The samples were analyzed using USEARCH (for details see Supplementary Methods).

### Differential abundance analysis

The operation taxonomic units (OTUs) present at the species level were evaluated for their differential presence across the patient groups. Initially, the R package, DESeq2^36,37^, was used to perform a likelihood ratio test with shrinkage to the base 2 log fold difference to determine whether an OTU had a significantly different normalized count in one of the four groups compared to the normalized count in the HPV-negative control. Subsequently, pairwise comparisons versus the HPV-negative control were performed using the Benjamini-Yekutieli false discovery adjustment (adjusted *P* < 0.05). A similar analysis was performed with adjustment for age, BMI (≤25, >25), and ethnicity (Hispanic, non-Hispanic).

### Quantitative real-time PCR analysis

Relative abundance of four *Lactobacillus* spp. was determined by quantitative real-time PCR analysis, performed on an Applied Biosystems QuantStudio6 Flex Real Time PCR System (Life Technologies, Grand Island, NY) using DNA extracted from vaginal swabs, TaqMan Assays specific for *L. crispatus*, *L. gasseri*, *L. iners, L. jensenii* and panbacterial 16S rRNA genes and TaqMan Vaginal Microbiota Amplification Control and TaqMan Fast Advanced Master Mix (Life Technologies). Relative abundances were calculated using a standard curve method and the 16S rRNA gene level as an internal standard.

### Statistical analyses

Differences in the demographic variables between patient groups were tested using analysis of variance model (ANOVA) for continuous variables and Fisher’s exact test for categorical variables. The difference in normal (≤4.5) versus abnormal pH levels (>4.5) between patient groups was tested using Fisher’s exact test, overall and adjusted for age and BMI using logistic regression. *Lactobacillus* relative abundance was classified as *Lactobacillus*-dominant (LD) versus non-*Lactobacillus*-dominant (NLD) based on a cut-point of 80%. This cut-point classified roughly equal number of patients in the LD (n = 51) and NLD (n = 49) groups. Differences across patient groups were tested using Fisher’s exact test. Comparison of the abundance of the four predominant *Lactobacillus* species was tested using the nonparametric Kruskal-Wallis test.

For the immune mediator data, the values above the detection limit were substituted with the maximum observation values of the target and values below the detection limit were substituted with 0.5 of the minimum detectable concentration provided in manufacturer’s instructions. The log transformation was applied to normalize the data. The difference in the concentration between the patient groups was tested using a linear mixed effects model where group was a fixed effect and replicate was the random effect. The β-coefficient and standard error were estimated to show the direction of association. If the overall difference was significant (*P* < 0.05), paired tests were performed with Tukey adjustment. Comparisons were adjusted for BMI, age and ethnicity in the linear mixed effects models. The β-coefficients therefore represent the expected change in each immune mediator for that patient group relative to the HPV-negative control for two women with the same values of the clinical covariates. Preliminary analyses demonstrated that there were statistically significant interactions between *Lactobacillus* dominance (LD) and the patient groups on the levels of the immune mediators (*P* < 0.05). Thus, whether there were significant differences between the severity groups depended on LD on non-*Lactobacillus*-dominance (NLD). For these immune mediators, comparison between the groups was performed separately for LD and NLD. To reduce concerns about multiple comparisons, we evaluated a previously described scoring system based on the levels of 7 cytokines. We assessed various scoring systems (e.g. 3/7, 4/7, 5/7, 6/7, 7/7) and computed overall *P* values using Fisher’s exact test (Supplementary Fig. S8A). Additionally, we tested for trend using logistic regression (treating the number of cytokines as a continuous variable). As confirmation, we performed a principal components analysis and assessed differences between the severity groups overall and after adjusting for age, BMI and ethnicity (Supplementary Fig. S8B). The overall assessment was based on ANOVA with pairwise comparisons adjusted using Tukey’s procedure. All analyses were performed using SAS 9.4.

## Electronic supplementary material

Supplementary Information

Supplementary Dataset
