# Supplementary material for: Linking cervicovaginal immune signatures, HPV and microbiota composition in cervical carcinogenesis in non-Hispanic and Hispanic women
Source: Sci Rep. 2018 May 15;8:7593. doi: 10.1038/s41598-018-25879-7 (PMC5954126; doi:10.1038/s41598-018-25879-7)
Supplement: Supplementary file 1 — Supplementary Information [file 41598_2018_25879_MOESM1_ESM.pdf]

Supplementary information

Linking cervicovaginal immune signatures, HPV and microbiota composition in cervical carcinogenesis in non-Hispanic and Hispanic women

Paweł Łaniewski[1], Dominique Barnes[2,3], Alison Goulder[1], Haiyan Cui[4], Denise J. Roe[4], Dana M. Chase[2,3,4,5,6], Melissa M. Herbst-Kralovetz[1,4,5,*]

[1]Department of Basic Medical Sciences, College of Medicine-Phoenix, University of Arizona, Phoenix, AZ, [2] Maricopa Integrated Health Systems, Phoenix, AZ, [3]Dignity Health St. Joseph's Hospital and Medical Center, Phoenix, AZ, [4]UA Cancer Center, University of Arizona, Tucson/Phoenix, AZ, [5]Department of Obstetrics and Gynecology, College of Medicine-Phoenix, University of Arizona, Phoenix, AZ, [6]US Oncology, Phoenix, AZ

* Correspondence:

Melissa M. Herbst-Kralovetz

Telephone: (602) 827-2247

Fax: (602) 827-2127

Email: mherbst1@email.arizona.edu

Supplementary datasets:

Dataset 1. Vaginal microbiota (VMB) profiles. Number of OTU reads for each sequenced sample.

Supplementary methods:

16S rRNA sequencing analysis. 16S rRNA sequencing analysis was performed by the Second Genome Inc. (San Francisco, CA). The V4 region of bacterial 16S rRNA operon was amplified from the genomic DNA obtained from vaginal swabs using fusion primers incorporating sequences for Illumina adapters (Illumina, San Diego, CA) and indexing barcodes. 98 samples met the post-PCR quantification minimum set by the Second Genome Inc., pooled equimolar and were advanced for purification and 250 cycles of sequencing on the MiSeq instrument (Illumina). Sequenced paired-end reads were merged using USEARCH and the resulting sequences were compared to an in-house strains database using USEARCH (usearch_global). All sequences hitting a unique strain with an identity >=99% were assigned a strain operation taxonomic unit (OTU). To ensure specificity of the strain hits, a difference of >=0.25% between the identity of the best hit and the second best hit was required (e.g. 99.75 versus 99.5). For each strain OTU one of the matching reads was selected as representative and all sequences were mapped by USEARCH (usearch_global) against the strain OTU representatives to calculate strain abundances. The remaining non-strain sequences were quality filtered and dereplicated with USEARCH. Resulting unique sequences were then clustered at 97% by UPARSE (de novo OTU clustering) and a representative consensus sequence per de novo OTU was determined. All non-strain sequences that passed the quality filtering were mapped to the representative consensus sequences to generate an abundance table for de novo OTUs. Representative OTU

sequences were assigned taxonomic classification via mothur's bayesian classifier, trained against the Greengenes reference database of 16S rRNA gene sequences clustered at 99%. All VMB profiles were inter-compared in a pairwise fashion to determine a dissimilarity score and store it in a distance dissimilarity matrix. Abundance-weighted sample pairwise differences were calculated using the Bray-Curtis dissimilarity. Bray-Curtis dissimilarity was calculated by the ratio of the summed absolute differences in counts to the sum of abundances in the two samples. The binary dissimilarity values were calculated with the Jaccard index. Principal Coordinate Analysis (PCoA) was used to graphically summarize the inter-sample relationships. Permutational Analysis of Variance (PERMANOVA) was utilized for finding significant differences among discrete categorical or continuous variables. In this randomization/Monte Carlo permutation test, the samples were randomly reassigned to the various sample categories, and the between-category differences were compared to the true between-category differences.

Supplementary tables:

Table S1. Vaginal pH significantly increases with severity of cervical neoplasm after adjustment for age and BMI. Statistical significance of association between vaginal pH (normal vs. abnormal) and patient group adjusted for age and BMI. *P* values calculated using Fisher's exact test overall and adjusted for age and BMI using logistic regression.

**Comparison**		***P* value**
**overall**		**0.003**
**pairwise**	Ctrl HPV- vs. Ctrl HPV+	0.20
	Ctrl HPV- vs. LGD	0.89
	Ctrl HPV- vs. HGD	**0.006**
	Ctrl HPV- vs. ICC	**0.02**
	Ctrl HPV+ vs. LGD	0.99
	Ctrl HPV+ vs. HGD	0.90
	Ctrl HPV+ vs. ICC	0.80
	LGD vs. HGD	0.95
	LGD vs. ICC	0.60
	HGD vs. ICC	0.98

Table S2. Vaginal pH, age and ethnicity are significantly correlated with VMB β-diversity. A PERMANOVA using distance matrices was performed for each variable to determine if they significantly contributed to the β-diversity of the samples.

**Variable**	***P* value**
Patient group (Ctrl HPV-, Ctrl HPV+, LGD, HGD, ICC)	0.165
Vaginal pH	**0.002**
Ethnicity	**0.017**
Age	**0.027**
BMI	0.149
HPV status	0.977

Table S3. List of exclusion criteria with verification methods.

Exclusion criteria	Verification method
Any skin condition, as determined by the physician, which could interfere with the conduct of the study or increase the risks to the test subject including any open or healing cuts/ incisions, abrasions, or broken skin in the testing area as determined by the gynecologist	Self-reported and pelvic exam
Currently menstruating	Self-reported and pelvic exam
Currently using or have used antibiotics, antifungals, topical steroids or antiviral (oral or topical in the testing areas) within 3 months prior to visit	Self-reported and medical record
Current or history of genital herpes	Pelvic exam and medical record
Vaginal infection (including candidal infection or bacterial vaginosis), vulvar infection, urinary tract infection, sexually transmitted infection (chlamydia, gonorrhea, trichomoniasis, genital herpes) vaginal or urethral discharge (current or in the past 3 weeks)	Self-reported and pelvic exam
Using lotion, powder, ointment, cream, perfume or oil on the skin in the testing areas region 48 hours prior to visit	Self-reported
Using douching substances, vaginal medications, vaginal suppositories, and feminine deodorant spray, wipes, or lubricants to the genital area within 48 hours prior to the visit	Self-reported
Sexual intercourse within 48 hours prior to the visit	Self-reported
Current treatment for any skin conditions on the testing area	Self-reported
Have bathed or have gone swimming within 4 hours prior the visit	Self-reported
Have shaved, waxed or used depilatory treatments in the testing area within 72 hours prior to the visit	Self-reported
Have smoked or consumed anything containing nicotine within 2 hours prior to visit	Self-reported
Type I or type II diabetes	Self-reported and medical record
Hepatitis or being HIV-positive	Medical record
Other medical conditions (such as an immunosuppressive condition), which in the investigator's opinion would compromise their participation	Medical record

Supplementary figures:

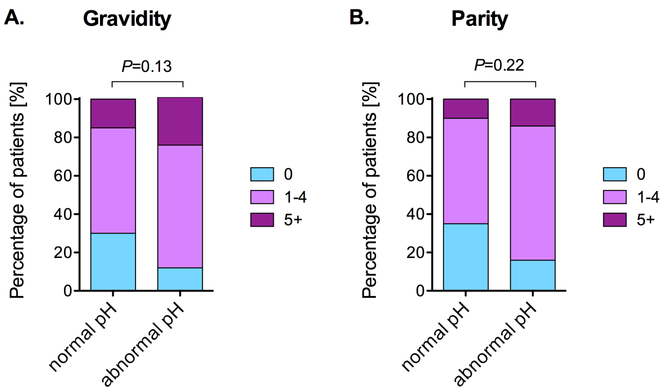

Figure S1. Vaginal pH is not significantly associated with gravidity or parity. Percentage of patients having 0, 1-4 and 5+ pregnancies (**A**) or live births (**B**). *P* values were calculated using Fisher's exact test.

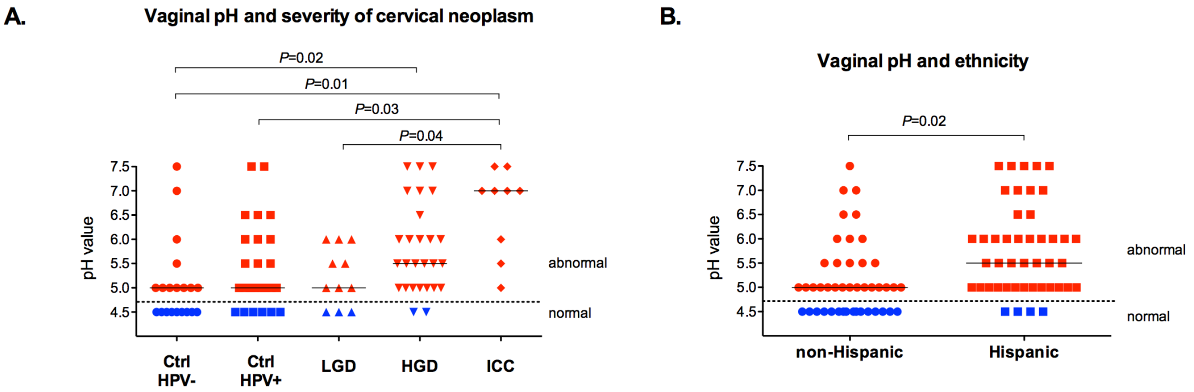

Figure S2. Vaginal pH significantly increases with severity of cervical neoplasm and is overall higher in Hispanic women. Vaginal pH levels in individual patients among the groups with different severity of cervical neoplasia (**A**) or ethnicities (**B**). A bar indicates a median. *P* values calculated using the Kruskal-Wallis test, with pairwise comparisons using Wilcoxon Rank Sum test.

A. Unweighted ordination

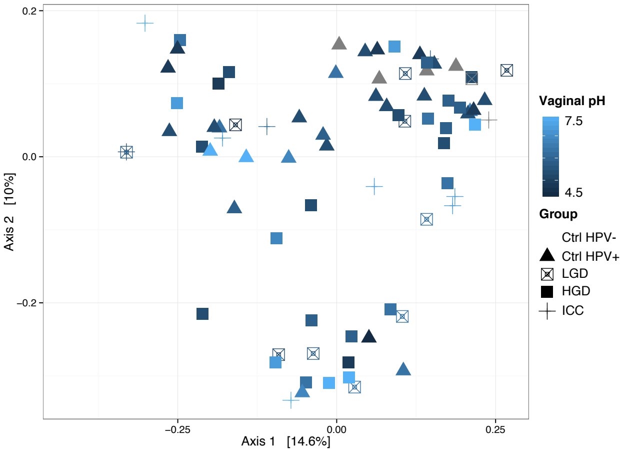

B. Weighted ordination

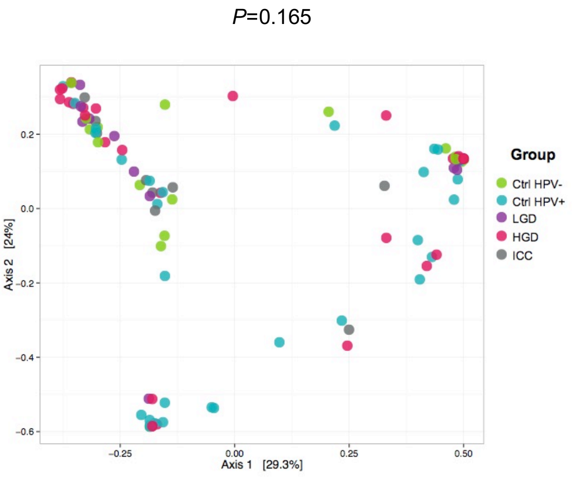

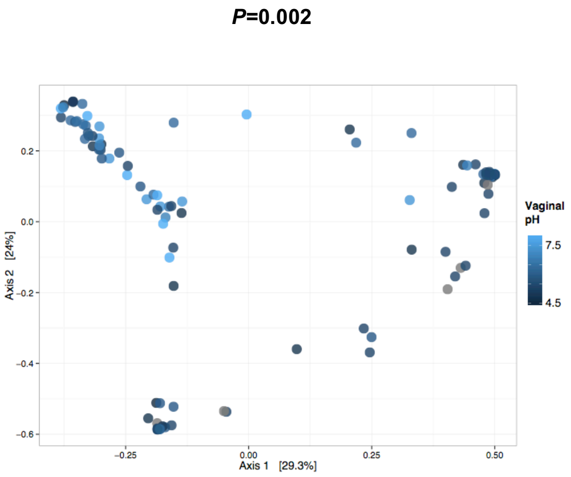

Figure S3. VMB samples do not cluster according to patient groups, but do cluster according to vaginal pH. Unweighted principal coordinate analysis (PCoA) of VMB samples using the Jaccard distance (**A**). The first two ordination axes accounted for 24.6% of sample variation. Weighted PCoA of VMB samples using the Bray-Curtis distance between VMB samples (**B**). The first two ordination axes accounted for 53.3% of sample variation. *P* value were calculated using PERMANOVA.

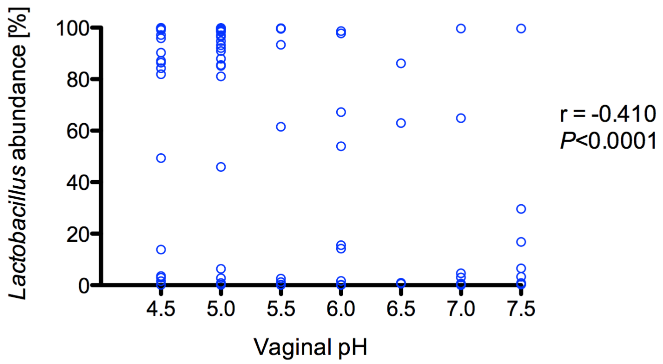

Figure S4. Vaginal pH level significantly correlates with *Lactobacillus* abundance. Spearman's correlation between vaginal pH level and relative abundance of *Lactobacillus* spp. n=93; critical value for Spearman's ranked correlation coefficient (r) = 0.204. *P* value was calculated using two-tailed test.

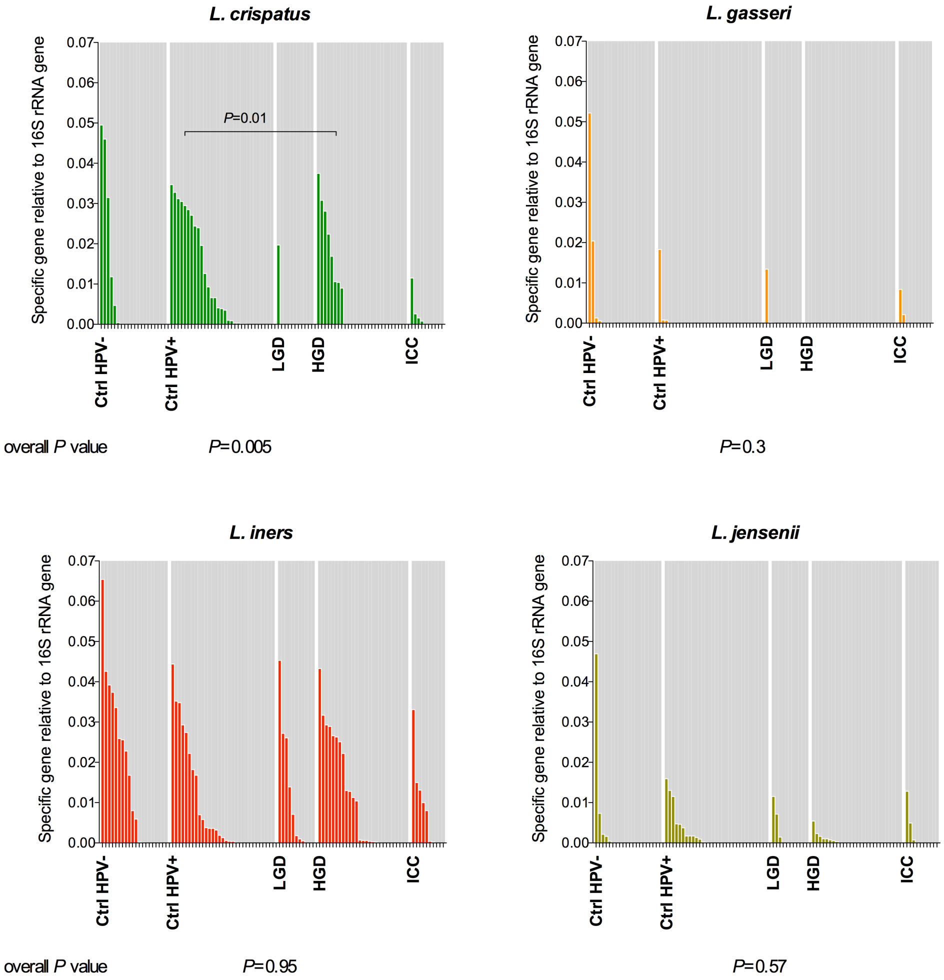

Figure S5. Abundance of *L. crispatus,* but not *L. iners, L. gasseri* or *L. jensenii* significantly differs across the groups. Abundance of four most frequent vaginal *Lactobacillus* spp. (*L. crispatus*, *L. gasseri*, *L. iners* and *L. jensenii*) was determined by quantitative real-time PCR arrays specific for each *Lactobacillus* sp. and pan-bacterial 16S rRNA gene. Relative abundance was extrapolated form standard curve and normalized to 16S rRNA. *P* values were calculated using Kruskal Wallis test.

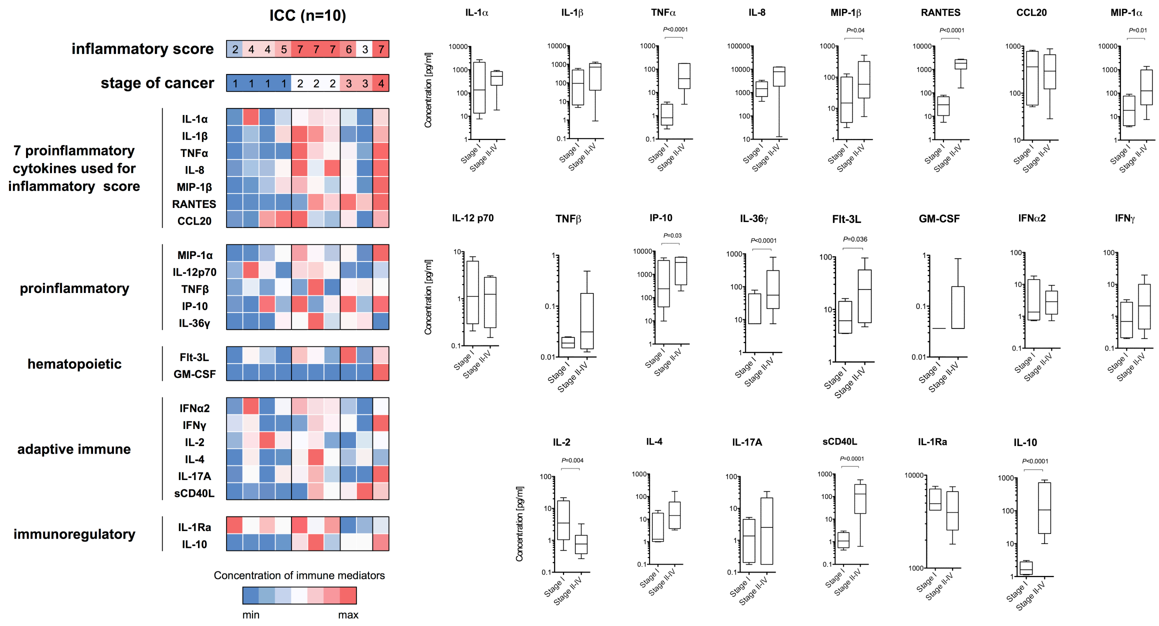

Figure S6. Levels of proinflammatory and chemotactic cytokines were significantly higher in stage II-IV ICC patients compared to stage I patients. Heat map reflects relative levels of immune mediators across ICC samples, stage of cervical cancer and inflammatory scores. Increasing brightness of red and blue indicate higher and lower concentration of each protein, respectively. Boxplot whiskers range between 10th and 90th percentiles, dots indicate outliers. *P* values were calculated using linear mixed effects models where group was the fixed effect and replicate was the random effect with Tukey adjustment.

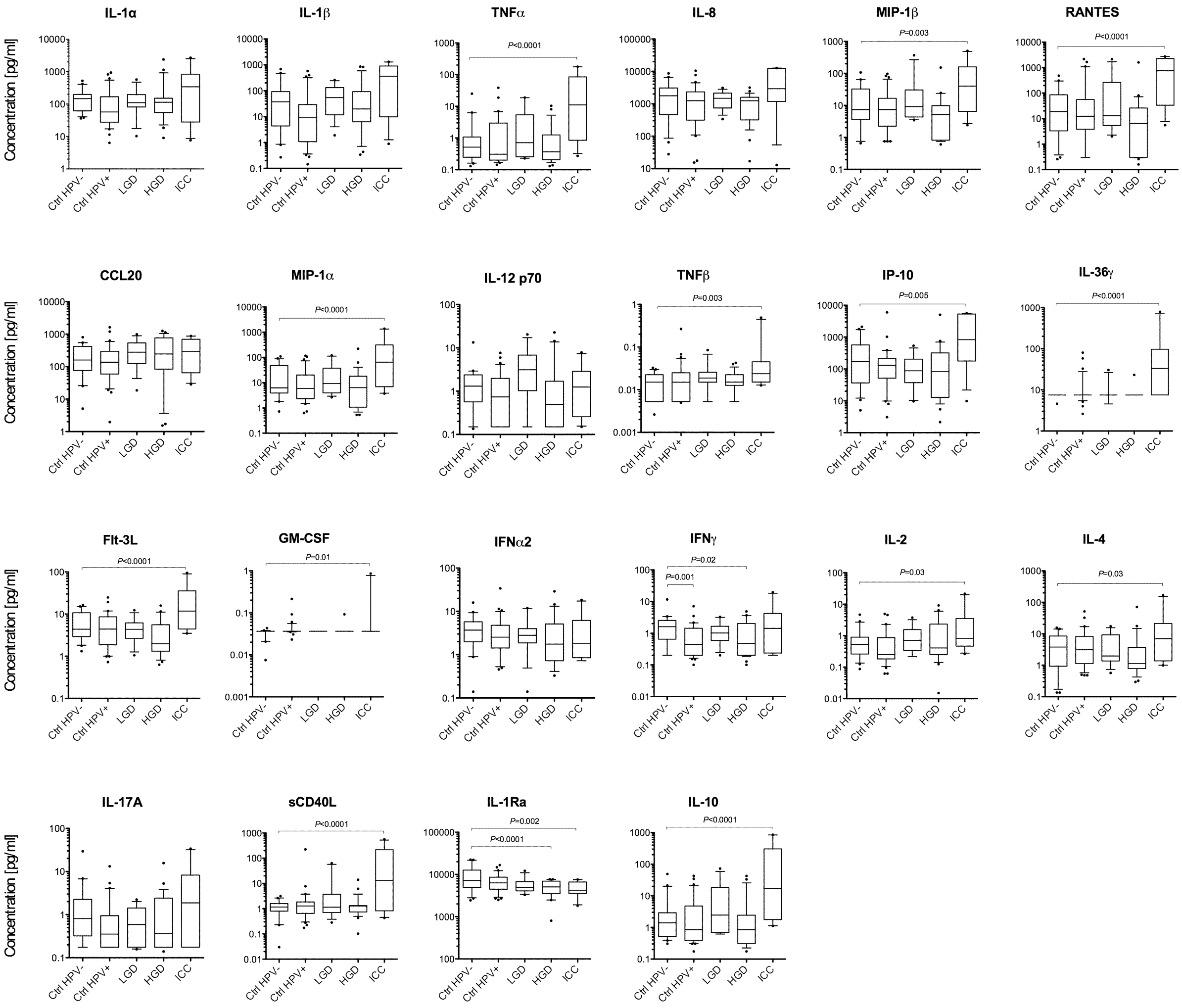

Figure S7. Levels of all tested immune mediators in cervicovaginal lavages among the groups. Boxplot whiskers range between 10th and 90th percentiles, dots indicate outliers. Capped lines indicate levels that were significantly altered compared to Ctrl HPV- group. *P* values were calculated using linear mixed effects models where group was the fixed effect and replicate was the random effect with Tukey adjustment.

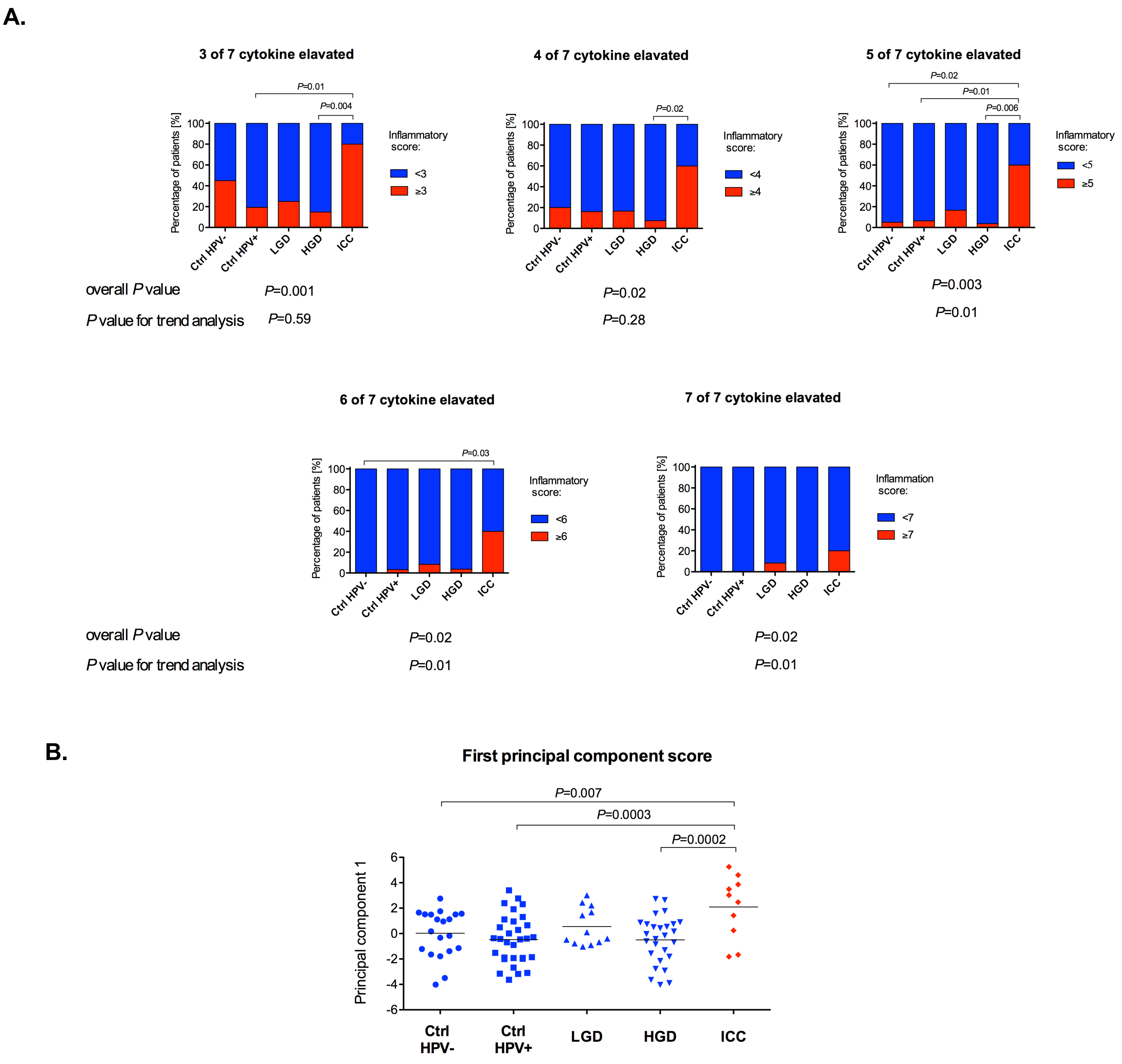

Figure S8. Comparison of scoring systems for evaluation of genital inflammation. Percentage of patients having at least 3/7, 4/7, 5/7, 6/7 or 7/7 of the following cytokines in the upper quartile: IL-1α, IL-1β, IL-8, MIP-1β, CCL20 (MIP-3α), RANTES, and TNFα (**A**). *P* values were calculated using Fisher's exact test, with the *P* value for the trend analysis calculated using a logistic regression model. First principal component scores in individual patients by the patient groups (**B**). *P* value calculated using analysis of variance, with pairwise comparisons using Tukey adjustment.

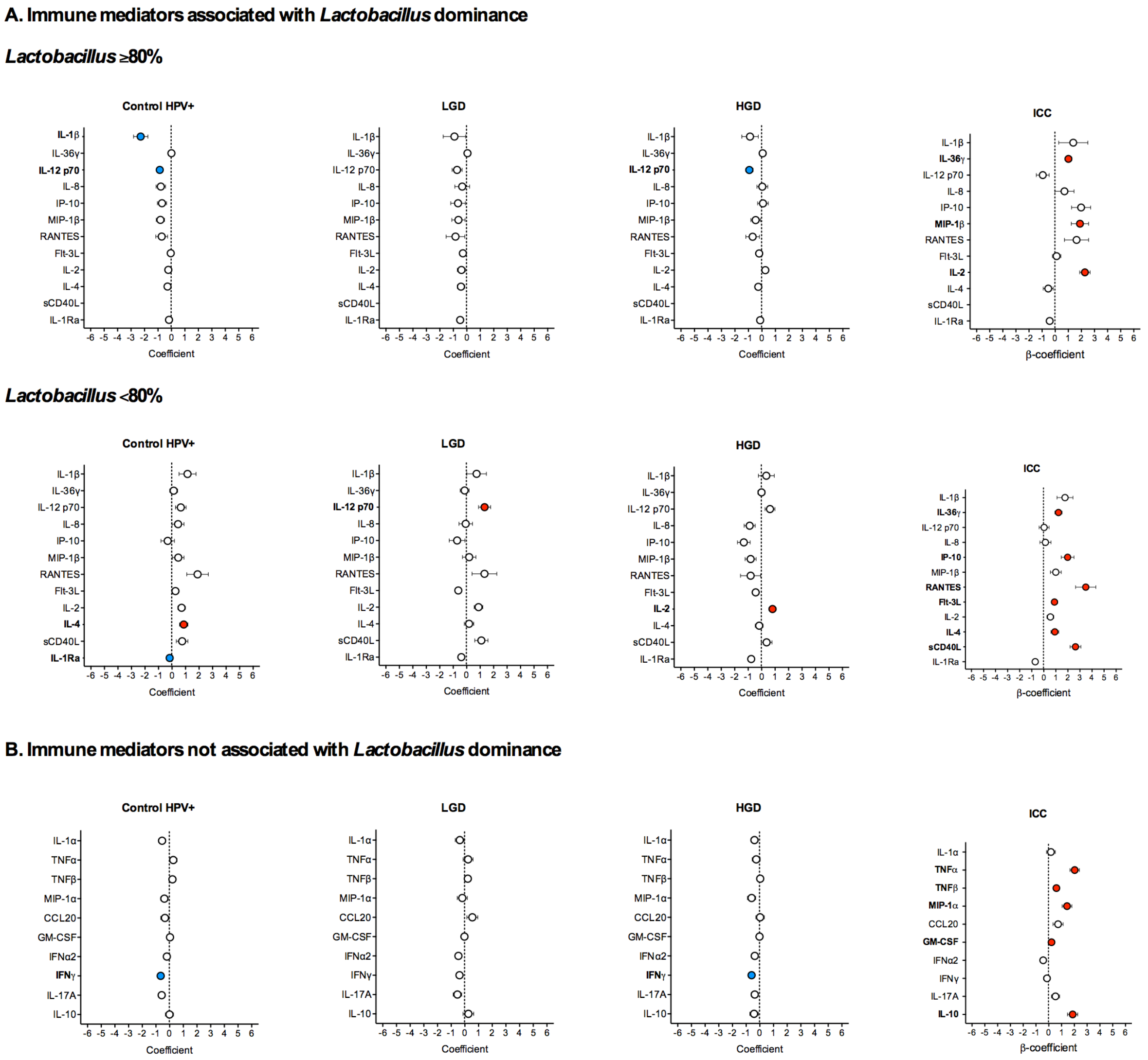

Figure S9. Unique immune signatures are significantly associated with ICC after adjusting for *Lactobacillus* dominance, age, BMI, and ethnicity. Panels show β-coefficients of immune mediators that were not associated with (**A**) or associated with *Lactobacillus* dominance (defined as ≥80% relative abundance) (**B**). Dots indicate β-coefficients of linear regression analysis; error bars represent standard error (SE). Red and blue dots indicate positive or negative associations, respectively, that were significant compared to Ctrl HPV- after adjusting for covariates (P<0.05).

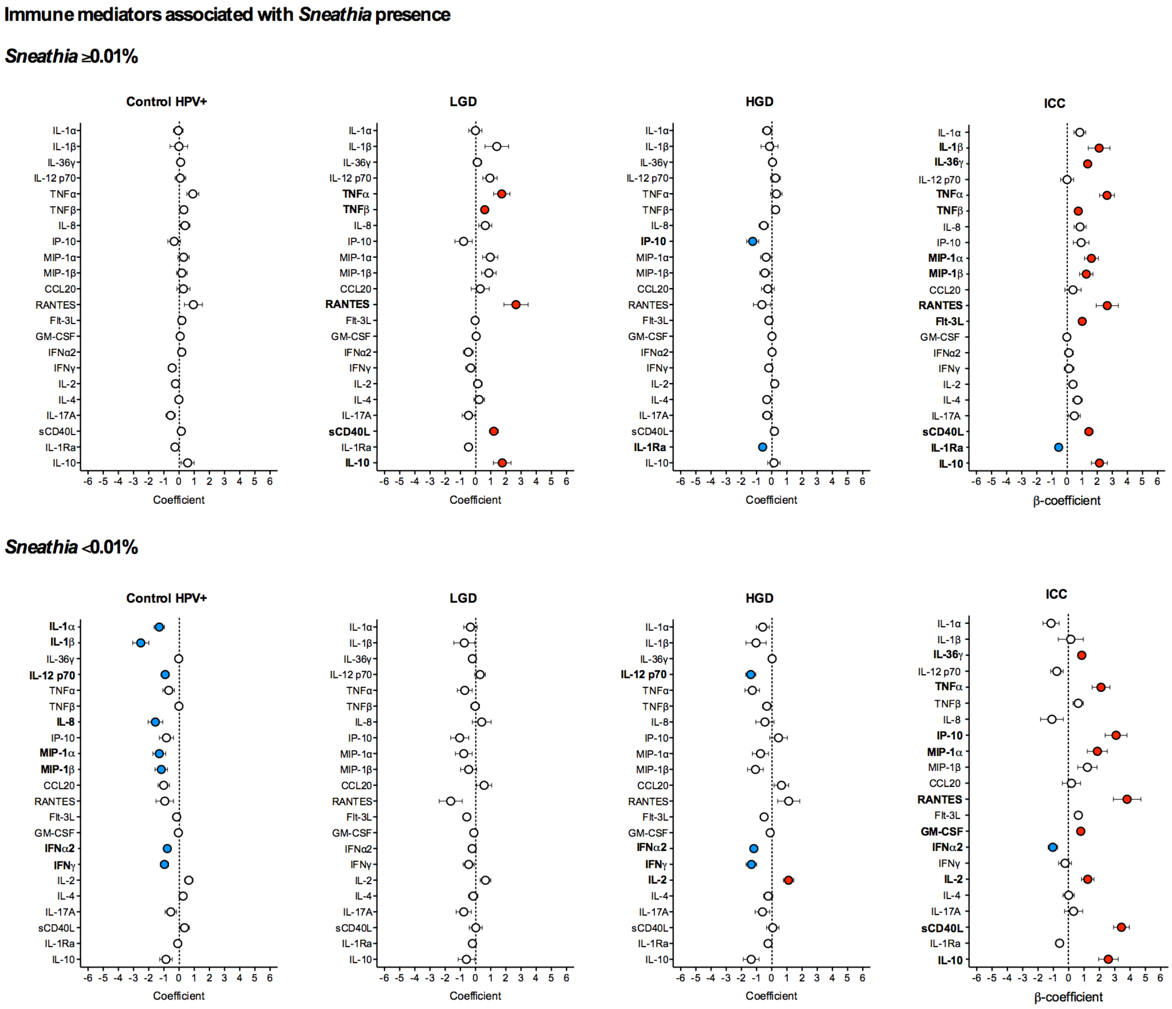

Figure S10. Unique immune signatures are significantly associated with ICC after adjusting for *Sneathia* presence, age, BMI, and ethnicity. Panels show β-coefficients of immune mediators that were associated with *Sneathia* presence (defined as ≥0.01% relative abundance). Dots indicate β-coefficients of linear regression analysis; error bars represent standard error (SE). Red and blue dots indicate positive or negative associations, respectively, that were significant compared to Ctrl HPV- after adjusting for covariates ($P<0.05$).
